# Supplementary material for: Burnout syndrome among healthcare workers during COVID-19 Pandemic in Accra, Ghana
Source: PLoS One. 2022 Jun 16;17(6):e0268404. doi: 10.1371/journal.pone.0268404 (PMC9202923; doi:10.1371/journal.pone.0268404)
Supplement: S1 Table — (DOCX) [file pone.0268404.s001.docx]

Table 1. Socio-demographic characteristics of participants

| **Socio-demographic characteristics** | **Clinical, n (%)**  **703 (55.62)** | **Non-Clinical, n (%)**  **561 (44.38)** | **p- value** |
| --- | --- | --- | --- |
| **Age in years: mean (± SD)** | 41.05±8.30 | 40.51±8.38 | 0.255 |
| **Sex of respondent** |  |  | 0.146 |
| Male | 317 (45.09) | 276 (49.20) |  |
| Female | 386 (54.91) | 285 (50.80) |  |
| **Marital status** |  |  | 0.393 |
| Single | 106 (15.01) | 99 (17.65) |  |
| Married | 428 (60.88) | 347 (61.85) |  |
| Divorced/Separated | 169 (24.04) | 115 (20.50) |  |
| **Having Children** |  |  | < 0.001 |
| Yes | 409 (57.75) | 399 (71.12) |  |
| No | 294 (41.82) | 162 (28.88) |  |
| **Highest educational level** |  |  | < 0.001 |
| Low | 0(0) | 33 (5.88) |  |
| Middle | 6 (0.85) | 304 (54.19) |  |
| High | 697 (99.15) | 224 (39.93) |  |
| **Range of net monthly income** |  |  | < 0.001 |
| Low | 251 (35.70) | 483 (37.79) |  |
| Middle | 392 (55.76) | 78 (66.13) |  |
| High | 60 (8.53) | 0 (0) |  |
| **Years of working** |  |  |  |
| 1-5 | 267 (37.98) | 227 (40.46) | < 0.001 |
| 6-10 | 133 (18.92) | 88 (15.69) |  |
| 11-15 | 23 (3.27) | 50 (8.91) |  |
| 16-20 | 1. 7.11) | 54 (9.63) |  |
| - 20 | 230 (32.72) | 142 (25.31) |  |
| **Level of resilience** |  |  | < 0.124 |
| Low | 166 (23.61) | 160 (28.52) |  |
| Normal | 271 (38.55) | 209 (37.25) |  |
| High | 266 (37.84) | 131 (23.35) |  |

Table 2. Job-profile of participants

| **Job-profile of participants** | **Clinical, n (%)**  **703 (55.62)** | **Non-clinical, n (%) 561 (44.38)** | **p-value** |
| --- | --- | --- | --- |
| **Facility of working** |  |  | < 0.001 |
| Primary | 149 (21.19) | 134 (23.89) |  |
| Secondary | 264 (37.55) | 142 (25.31) |  |
| Tertiary | 290 (41.25) | 285 (50.80) |  |
| **Service Area** |  |  | < 0.001 |
| Highly dependent unit | 125 (17.78) | 51 (9.09) |  |
| Stable in-patients | 287 (40.83) | 177 (31.55) |  |
| Out-patient | 264 (37.55) | 0 (0) |  |
| No contact with patients | 27 (3.84) | 277 (49.38) |  |
| Management | 0 (0) | 56 (9.98) |  |
| **Common shift for past 6 months** |  |  | < 0.001 |
| Night | 269 (38.26) | 332 (59.18) |  |
| Afternoon | 228 (32.43) | 146 (26.02) |  |
| Morning | 206 (29.30) | 83 (14.80) |  |
| **Additional Jobs** |  |  | < 0.001 |
| Yes | 339 (48.22) | 205 (36.54) |  |
| No | 364 (51.78) | 356 (63.46) |  |
| **Intentions to leave job** |  |  | < 0.234 |
| Yes | 341 (48.51) | 291 (51.87) |  |
| No | 362 (51.49) | 270 (48.13) |  |
| **Job support** |  |  | < 0.001 |
| Yes | 249 (35.42) | 258 (45.99) |  |
| No | 454 (64.58) | 303 (54.01) |  |
| **Job control** |  |  | 0.073 |
| Yes | 309 (43.95) | 275 (49.02) |  |
| No | 394 (56.05) | 286 (50.98) |  |
| **Job satisfaction** |  |  | < 0.001 |
| Yes | 330 (46.94) | 215 (38.32) |  |
| No | 373 (53.06) | 346 (61.68) |  |
| **Perceived high workload** |  |  | < 0.001 |
| Yes | 110 (15.65) | 150 (26.74) |  |
| No | 593 (84.35) | 411 (73.26) |  |
