# Supplementary material for: Burnout syndrome among healthcare workers during COVID-19 Pandemic in Accra, Ghana
Source: PLoS One. 2022 Jun 16;17(6):e0268404. doi: 10.1371/journal.pone.0268404 (PMC9202923; doi:10.1371/journal.pone.0268404)
Supplement: Attachment — Submitted filename: PLOS ONE paper review 7.docx [file pone.0268404.s003.docx]

**PONE-D-21-24220**

Burnout syndrome among health workers in human resource-constrained settings in urban Ghana: socio-demographic and job-related predictors

**Comments:**

**Ethics statement:** The authors stated that: the data in this paper is part of study titled “burnout, metabolic syndrome and predisposition to non-communicable diseases”. Also, this was mentioned in page 9 , second paragraph. Was the original study published? If yes, you need its reference?

**Abstract:** It is very long and should be rewritten. As per PLOS instructions to authors: authors should summarize the most important results and their significance and not exceed 300 words.

**Methods:** Explain in more details’ inclusion and exclusion criteria of the participants.

What was the date and period of the study?

Page 6 paragraph 5: the authors stated that: “All participants who took part in this study were not directly involved in caring for patients with COVID-19……….” How about clinicians working in ER and ICU ….and other department caring for patients with COVID-19. Please clarify.

Explain” Veronica bucket” as a mechanism for hand washing originating in Ghana and readers are not familiar with it.

Under instrument: How long did it take to complete the questionnaires?

Level of Resilience: What were the scores for low, normal and high levels showed in Table 1?

Burnout syndrome: What are the scores in Masalah Burnout Inventory to indicate “no burnout” versus “burnout”? in other words to diagnose burnout syndrome? Furthermore, was the latter classified into mild, moderate, and severe? The authors stated that “Participants with high score in EE and depersonalization…” what are the levels of high, moderate, and low scores? Also, these were mentioned under Table 4.

How were the data extracted?

**Results:**

Authors should prove that all clinical and non-clinical participants were comparable in all possible confounding factors related to the outcome. Instead of reporting the total number column in Table 1, do statistical analysis to see if there were statistically significant between both groups.

What was the range of the participants’ age?

Under Prevalence of burnout among participants page 14: the authors stated “….. compared to the clinical participants, the non-clinical participants had a significantly higher prevalence of burnout” What was the p-value?

**Tables:** there are 10 Tables, large numbers. I suggest delete some of them such as Table 3 and instead mention the findings in the text. Table 4 and Table 5 can be merged under one Table and the same for Table 7 with 9 and 8 with 10. Or just keep one of each similar Table and mention the positive finding of the other in the text.

Table 2: What were the definitions of night, afternoon, and morning work shifts? What were job support and job control and how did they measure them? Explain these in the text.

**Discussion:** on page 22, second paragraph authors sated that “The finding of high burnout rate could be because of the reduced resources resources……………………. inadequate preparedness of health workers to handle emerging disease….” These were assumption and were not investigated in this study. I suggest deleting this paragraph.

**Limitation of the study:** explain in logical ways. This study was susceptible to survivor bias because it

assesses prevalence rather than incident cases. It is also likely that all the scores were subjected to reporting bias, since the data here were collected through a questionnaire.

**References:** PLOS uses “Vancouver” style. The manuscript presents several references which were outdated. The references should be revised, updated, and should follow the guidelines of the journal.
