# Supplementary material for: Burnout syndrome among healthcare workers during COVID-19 Pandemic in Accra, Ghana
Source: PLoS One. 2022 Jun 16;17(6):e0268404. doi: 10.1371/journal.pone.0268404 (PMC9202923; doi:10.1371/journal.pone.0268404)
Supplement: Attachment — Submitted filename: Response to Reviewers.docx [file pone.0268404.s004.docx]

University of Ghana

College of Health Sciences

School of Public Health

Department of Social & Behavioural

Sciences

4^th^ February, 2022

The Editor

PLos One

Dear Sir/Madam,

**RESPONSE TO MANUSCRIPT COMMENTS**

We are grateful that you considered our manuscript for review. We have provided a point by point response to each observation made by the reviewer.

**Authors’ general comments**

We are most grateful that you spent time within your important schedule to review and make insightful remarks regarding our manuscript. Your contributions and views are generally appreciated. We have incorporated the comments as you indicated within the manuscript.

**Editor’s comments**

**The concerns of the editor are outlined below:**

-Revising the title of the study to include COVID-19

-Stating the research questions clearly and in detail

-Paying more attention to the methods section (i.e., sample size calculation, inclusion/exclusion criteria, …)

-Paying more attention to the definitions of some variables and the process of categorizing them. -Also PLS describe how the categories of burnout were created.

-Merging tables and revising them according to the research questions.

-Discussing other potential risk factors of occupational burnout during the COVID-19 pandemic

-Discussing potential sources of bias in the study

-Paying more attention to the usage of acronyms

**Author’s responses to Editor’s comments**

- The title of the manuscript has been revised to include COVID-19 as recommended by the editor (title page ie page 1)
- The research question has been stated clearly and in detail in page 4 .of the manuscript
- The authors have paid more attention to the methods section particularly stating the inclusion and erxclusion criteria in page 5 and stating the methods for deriving the sample size in page 5.
- The authors have clearly stated how the variables such as level of resilience, burnout among others were derived/defined on page 7 and 8 of the manuscript.
- The authors have merged the tables as recommended and reduced the number of tables from 11 tables to 6 tables.
- Other potential risk factors for burnout during the COVID-19 period have been discussed in the limitations of the study on page 23 with supporting references.
- The potential sources of bias have been discussed in the limitations of the study on page 23.
- The number of acronmys have been reduced and attention given to those in the manuscript as recommended.

**Additional Editor Comments:**

**The concerns of the additional editor are outlined below:**

Thank you for working on burnout during COVID-19 pandemic. This is an interesting and timely study. The authors collected data on several important variables related to burnout but the results of the study have not been presented in a satisfactory manner. In my opinion, the authors should define the research questions clearly at the beginning and focus on them in results section. For example, if the main aim of the study is to investigate predictors of burnout then there is no need to present the socio demographic data according to clinical/nonclinical state of the participants. Instead, they should consider clinical/nonclinical state of the participants as an independent variable in the tables. If the authors aimed to compare the level of burnout between clinician and non-clinician individuals, they should include p-value for comparisons in the tables.
As the authors mention in the manuscript, the fear of contracting COVID-19 in the workplace could lead to burnout. Therefore, I strongly recommend that the authors include 'COVID-19' in the title of their paper. The title proposed by one of the reviewers seems more interesting and appropriate with regard to the content of this study. I also recommend that the authors provide some information in the introduction section on the situation of the country during the study period in terms of COVID-19 infection/death rates.
-Background and Conclusions in the abstract should be summarized

-The period of the study should be mentioned in the abstract.
-The inclusion/exclusion criteria should be clearly stated.
-Tables should be clearly laid out with no need for supplementary information. PLS spell out the abbreviations in the footnotes of tables.
-Table 3 is suitable for a validity study of a scale.
-The number of tables is unnecessarily high. Tables should be merged according to the research questions. If the authors’ aim is to present data for predictors of burnout then they should merge:
-tables 1 and 2
-tables 3, 4, 5, and 6
-tables 7 and 8
-tables 9 and 10.
I hope the authors take note of all my comments and improve the quality of the manuscript.

**Authors response to additional editor’s comments**

- The results have being re-organised and p-values for comparison of the socio-demographic and job-related factors have been present in Tables 1 and 2 on pages 10 and 12
- COVID-19 has been included in the title of the study as recommended
- Information on the COVID-19 situation in Ghana during the study period has been provided in the last paragraph of the introduction on page 4 of the manuscript
- The background and conclusion in the abstract have been summarized as recommended.
- The period of the study have been mentioned in the abstract (methods of abstract)
- The inclusion and exclusion criteria have been stated in the methods of the manuscript on page on page 5 (selection of participants and data collection).
- Tables have been clearly laid out in the manuscript with appropriate footnotes
- Table 3 has been taken out as recommended
- The number of tables have been reduced and those mergers recommended have been done.

**Reviewer’s comments**

Reviewer #1: I would like to thank you for inviting me to review this manuscript. In the present study, a cross-sectional study design was used to investigate the prevalence of burnout and its associated factors among health workers in human resource-constrained settings in urban Ghana. In my view, although the research idea is highly interesting, the study could be more precise. The following comments would be helpful to enhance the quality of the study to be endorsed by the scientific community.
1. Regardless of the results obtained from the model, it is crucial to emphasize that accurate predictions cannot be guaranteed by cross-sectional study. Rather, development of prediction models is based on cohort study. Thus, prediction models resulting from cross-sectional designs can be misleading. Therefore, it is necessary to consider this point in the interpretation of the results of this study. I recommend that the authors use the word association (or relationship) instead of prediction in the whole article.
2. The title of the article does not reflect the content appropriately. What do you think about the following title?
“Burnout among clinical and non-clinical health workers in Ghana during the COVID-19 pandemic: The roles of socio-demographic and job-related factors”
3. In the “limitations of the study” section, I suggest the authors to add some brief consideration on other potential risk factors of occupational burnout (e.g., grief caused by multiple losses, personal characteristics, and limited supportive resources to handle workload) during the COVID-19 pandemic with appropriate references (see doi: 10.1177/20101058211040575; doi: 10.3889/oamjms.2020.5502; doi: 10.1007/s12144-020-01292-0).
4. The overall conclusion does not rise to the level of conceptual advance. The “conclusion” section should be briefly described the implications of the study, followed by recommendations for future studies.
5. In page 25, please replace “Afulani et al. (2021a)” with “Afulani et al. [2].”
6. Please review the manuscript for use of the acronyms. In addition, define the acronyms separately in the abstract and introduction before using them in other sections of the manuscript. Moreover, the number of acronyms in the article is so large which makes it hard for the reader to follow. Please minimize the use of acronyms in all parts of the article, particularly in the abstract section.
7. Please add acronyms in the footnotes of tables.

**Authors’ response to reviewer 1**

-The authors have used association/relationship throughout the entire article as recommended

- The title of the manuscirpt has been modified as recommended.

- The other risk factors of occupational burnout have been stated in the limitation section as recommended.

- The recommendeations on the conclusion section have been effected

- The suggestions on page 25 regarding the reference has been done

- the number of acronyms have been reduced and those used have been clearly defined

**Reviwer no. 2 comments**

**Reviewer #2:** Title of manuscript need revision in line with objective of study
in abstract, it should no more than 250 word
result need to revise
conclusion should be concise
in introduction
literature review should be recent and relevant with objective of study
rational of study should be strong
in method section
study setting should be explained more
sample size need explain how to calculate which software and parameter
study variable should be explain
exclusion criteria should be explain
study tool should be explain and reliability and validity should be include
statistical analysis should be revised and relevant statistical test should be apply

result of study should be revised according to revise test

in discussion need to revised with compare of relevant studies
limitation of study and bias of study should be add
references need to revise with latest after 2019

**Authors’ responses to reviewer no.2’s comments**

- The title of the manuscript has been revised in line with the objective of the study
- The results has been revised as recommended and number of tables reduced
- The conclusion has been made concise as recommended
- In the introduction, the literature used has been revised to more recent and rational for the study stated as recommended
- In the method section, the method in which the sample size was derived has been stated and study setting explained further. Also, the information on the study tool and their relevant validity has been stated. Further, the appropriate statistical approaches have been used to anaklyse the data.
- The results have been revised and this was used to revise the text.
- The disucssions have been compared with other relevant studies
- References have been revised and papers after 2019 inckuded as recommended.

**Reviewer no.3 comments**

**PONE-D-21-24220**

Burnout syndrome among health workers in human resource-constrained settings in urban Ghana: socio-demographic and job-related predictors

**Comments:**

**Ethics statement:** The authors stated that: the data in this paper is part of study titled “burnout, metabolic syndrome and predisposition to non-communicable diseases”. Also, this was mentioned in page 9 , second paragraph. Was the original study published? If yes, you need its reference?

**Abstract:** It is very long and should be rewritten. As per PLOS instructions to authors: authors should summarize the most important results and their significance and not exceed 300 words.

**Methods:** Explain in more details’ inclusion and exclusion criteria of the participants. What was the date and period of the study?

Page 6 paragraph 5: the authors stated that: “All participants who took part in this study were not directly involved in caring for patients with COVID-19..........” How about clinicians working in ER and ICU ....and other department caring for patients with COVID-19. Please clarify.

Explain” Veronica bucket” as a mechanism for hand washing originating in Ghana and readers are not familiar with it.

Under instrument: How long did it take to complete the questionnaires?

Level of Resilience: What were the scores for low, normal and high levels showed in Table 1?

Burnout syndrome: What are the scores in Masalah Burnout Inventory to indicate “no burnout” versus “burnout”? in other words to diagnose burnout syndrome? Furthermore, was the latter classified into mild, moderate, and severe? The authors stated that “Participants with high score in EE and depersonalization...” what are the levels of high, moderate, and low scores? Also, these were mentioned under Table 4.

How were the data extracted?

**Results:**

Authors should prove that all clinical and non-clinical participants were comparable in all possible confounding factors related to the outcome. Instead of reporting the total number column in Table 1, do statistical analysis to see if there were statistically significant between both groups.

What was the range of the participants’ age?

Under Prevalence of burnout among participants page 14: the authors stated “..... compared to the clinical participants, the non-clinical participants had a significantly higher prevalence of burnout” What was the p-value?

**Tables:** there are 10 Tables, large numbers. I suggest delete some of them such as Table 3 and instead mention the findings in the text. Table 4 and Table 5 can be merged under one Table and the same for

Table 7 with 9 and 8 with 10. Or just keep one of each similar Table and mention the positive finding of the other in the text.

Table 2: What were the definitions of night, afternoon, and morning work shifts? What were job support and job control and how did they measure them? Explain these in the text.

**Discussion:** on page 22, second paragraph authors sated that “The finding of high burnout rate could be because of the reduced resources resources......................... inadequate preparedness of health workers to handle emerging disease....” These were assumption and were not investigated in this study. I suggest deleting this paragraph.

**Limitation of the study:** explain in logical ways. This study was susceptible to survivor bias because it assesses prevalence rather than incident cases. It is also likely that all the scores were subjected to

reporting bias, since the data here were collected through a questionnaire.

**References:** PLOS uses “Vancouver” style. The manuscript presents several references which were outdated. The references should be revised, updated, and should follow the guidelines of the journal.

**Authors’ responses to reviewer 3**

- There is no publication yet from the study and this has been stated on page 9 on ethics In the manuscript.
- The length of the abstract has been reduced to less than 300 words as recommended
- The inclusion and exclusion criteria has been stated on page 5 of the manuscript.
- The date and period of the study has been stated in the abstract and on the last paragraph of page 6 of the manuscript.
- The suggestions on page 6 of the manuscript have been effected.
- The clarifications on veronica bucket has been done.
- The duration for filling the instrument has been stated in the second sentence under instrument on page 7.
- The scores under level of resilience have been stated on page 8 of the manuscript.
- The determination of burnout and the scores on the elements of the burnout syndrome have been stated on page 8 under burnout syndrome.
- How the data was extracted is provided under data analysis on page 8 of the manuscript.
- The comparison of the study participants have been made under table 1 as suggested.
- The age range for the participants has been stated in page 10 of the manuscript.
- The p-value for the prevalence of burnout has been provided on page 13 of the manuscript as recommended.
- The tables have been merged as recommended by the reviewer
- The definitions for night, afternoon and morning shifts have been defined on page 11 of the manuscript
- Also, the definitions for job control and job support have been provided on page 11 of the manuscript.
- The recommendations on the discussions, limitations and references have been effected .

**Conclusions**

- We do hope that the corrections we have made in the manuscript meet the demands of the reviewer.

Yours sincerely,

Kennedy Dodam Konlan
