# Supplementary material for: Burnout syndrome among healthcare workers during COVID-19 Pandemic in Accra, Ghana
Source: PLoS One. 2022 Jun 16;17(6):e0268404. doi: 10.1371/journal.pone.0268404 (PMC9202923; doi:10.1371/journal.pone.0268404)
Supplement: Attachment — Submitted filename: Response to Reviewers.docx [file pone.0268404.s005.docx]

University of Ghana

College of Health Sciences

School of Public Health

Department of Social & Behavioural

Sciences

23^rd^ March, 2022

The Editor

PLos One

Dear Sir/Madam,

**RESPONSE TO MANUSCRIPT COMMENTS**

We are grateful that you considered our manuscript for review. We have provided a point by point response to each observation made by the reviewer.

**Authors’ general comments**

We are most grateful that you spent time within your important schedule to review and make insightful remarks regarding our manuscript. Your contributions and views are generally appreciated. We have incorporated the comments as you indicated within the manuscript.

**Editor’s comments**

**The concerns of the editor are outlined below:**

-Revising the title of the study

- The results in the abstract should be presented together with statistical measures such as OR, p-value or CI.

-Stating the research questions clearly in both the text and the abstract

-Statistical tests performed need to be stated in the methods section of the abstract.

- Providing useful information about the settings of the study is important

- Merging tables 1-2 and revising them according to the research questions.

- Bias and external validity of study should be described

**Author’s responses to Editor’s comments**

- The title of the manuscript has been revised as recommended by the editor (title page ie page 1)
- The results in the abstract have been modified to include statistical measures such as OR, p-value or CI as recommended (page 2).
- The research question has been clearly stated in both the abstract and text as recommended.
- The statistical tests that were performed have been stated in the methods section of the abstract (page 2).
- The authors have provided additional information on the setting of the study has been provided on page 5 of the manuscript as suggested.
- Tables 1 and 2 have been merged into a single table as recommended.
- The bias and external validity of the study have been explained on page 23 as recommended.

**Additional Editor Comments:**

**The concerns of the additional editor are outlined below:**

-Socio-demographic and job-profiles of participants should be presented in a single table covering all the sample members.

-The title proposed by one of the reviewers seems more informative and appropriate with regard to the contents of the study: “burnout syndrome among healthcare workers during COVID-19 pandemic”

-The remarks on “Aims” in both the abstract and the text are in need of revision.

-Limitations should be included in the abstract.

I hope the authors take note of all my comments and the comments raised by reviewers and improve the quality of the manuscript.

**Authors’ response to additional editor’s comments**

- The results have being re-organised and Tables 1 and 2 have ben merged as suggested.
- The title of the study has been revised as suggested.
- The aim has been revised as recommended
- The limitation has been written properly and included in the abstract.

**Reviewer’s comments**

Reviewer #2:

-Title of study need to revise as burnout syndrome among Healthcare workers during COVID-19 pandemic

-in abstract method section need to revise as which statistical test apply,

-in result section finding should be mentioned in numbers

-Rational of study should be strong in the introduction section

- study setting need to explain more as how many patients coming to selected hospitals daily and how many doctors available

-sample size need to re-calculate

- chi square test not appropriate test to determine the association it need to apply regression test

-bias of study should be explain external validity of study should be explain.

**Authors’ response to reviewer 2**

-The title of the study has been revised as suggested.

- The statistical analysis that was done has been stated in the methods section of the abstract.

- The results section has been modified and specific numbers have been mentioned as recommended.

- The rationale for the study has been well stated in the introduction.

-The study settings has been explained further as recommended

- The rationale for the sample size has been explained.

- Regression analysis has been added in addition to the chi-square test.

-Bias and external validity of the study has been explained in the limitations of the study.

**Conclusions**

- We do hope that the corrections we have made in the manuscript meet the demands of the reviewer.

Yours sincerely,

Kennedy Dodam Konlan
