# Supplementary material for: Burnout syndrome among healthcare workers during COVID-19 Pandemic in Accra, Ghana
Source: PLoS One. 2022 Jun 16;17(6):e0268404. doi: 10.1371/journal.pone.0268404 (PMC9202923; doi:10.1371/journal.pone.0268404)
Supplement: Attachment — Submitted filename: Response to Reviewers.docx [file pone.0268404.s006.docx]

University of Ghana

College of Health Sciences

School of Public Health

Department of Social & Behavioural

Sciences

29^th^ March, 2022

The Editor

PLos One

Dear Sir/Madam,

**RESPONSE TO MANUSCRIPT COMMENTS**

We are grateful that you considered our manuscript for review. We have provided a point by point response to each observation made by the reviewer.

**Authors’ general comments**

We are most grateful that you spent time within your important schedule to review and make insightful remarks regarding our manuscript. Your contributions and views are generally appreciated. We have incorporated the comments as you indicated within the manuscript.

**Editor’s comments**

**The concerns of the editor are outlined below:**

Specifically:

- Appropriate statistical tests as noted by the reviewer should be performed.

- The percentage of burnout and how it is calculated should be clarified and presented in detail.

**Author’s responses to Editor’s comments**

- We have used the appropriate statistical test appropriate for the data that was collected. The outcome variable in this study was burnout which was classified as a categorical variable and hence logistic regression been appropriate for the analysis. Linear equations regressions as suggested by the reviewer is not appropriate when dealing with a categorical outcome variable.
- The percentage of burnout and how it was calculated has been clarified and presented in page 13 of the revised manuscript.

**Additional Editor Comments:**

**The concerns of the additional editor are outlined below:**

- In page 9 the authors state, “Participants with high scores on emotional exhaustion and depersonalization as well as low scores on personal accomplishment sub-scales were diagnosed as having burnout [24, 26].” But the authors’ criteria for designating a participant as having experienced burnout has not been clearly argued and justified and it seems arbitrary and inconsistent. The authors should determine the percentage of participant who experienced burnout.

- No information is presented regarding the number of participants with high scores on emotional exhaustion and depersonalization and low scores on personal accomplishment sub-scales. I think the authors should start with calculating the total score for this scale and then use it in linear regression analysis.

- PLS do not repeat the results both in text and in tables

**Authors’ response to additional editor’s comments**

- The authors have provided the diagnostic criterion for burnout on page 9 of the revised manuscript and supported same by literature. And the percentage of those with burnout stated on page 13 of the revised manuscript as requested.
- Information on participants with high scores on emotional exhaustion, depersonalization and personal accomplishment have been presented on page 13 of the revised manuscript (Table 2).
- Respectfully, the data for the study particularly the outcome variable (burnout) was categorical and thus not practicable to use linear regression analysis as argued. Linear regression analysis is used when the output or the dependent variable is continuous. Such models are used for risk assessment and predictions and this was not the focus of this study. We thus used the logistic regression analysis for our categorical outcome variable.
- It is thus properly so that we used logistic regression models in our analysis since that is appropriate for categorical outcome variables.
- We have ensured no repeat of results in text and tables.

**Reviewer’s comments**

Reviewer #2:

- It need to revise analysis

-Linear equations regression should apply.

-limitations of study should be add

-Discussion need revised

**Authors’ response to reviewer 2**

-We have revised the analysis as suggested

-Burnout in this study was classified as a categorical variable hence linear equation regression analysis cannot be applied as suggested.

-Limitations of the study have been added in the discussion section on page 24 of the revised manuscript.

-Discussions have been revised appropriately

**Conclusions**

- We do hope that the corrections we have made in the manuscript meet the demands of the editor and reviewer.

Yours sincerely,

Kennedy Dodam Konlan
