# Supplementary material for: Burnout syndrome among healthcare workers during COVID-19 Pandemic in Accra, Ghana
Source: PLoS One. 2022 Jun 16;17(6):e0268404. doi: 10.1371/journal.pone.0268404 (PMC9202923; doi:10.1371/journal.pone.0268404)
Supplement: Attachment — Submitted filename: Response to Reviewers.docx [file pone.0268404.s007.docx]

University of Ghana

College of Health Sciences

School of Public Health

Department of Social & Behavioural

Sciences

9^th^ April, 2022

The Editor

PLos One

Dear Sir/Madam,

**RESPONSE TO MANUSCRIPT COMMENTS**

We are grateful that you considered our manuscript for review. We have provided a point by point response to each observation made by the reviewer.

**Authors’ general comments**

We are most grateful that you spent time within your important schedule to review and make insightful remarks regarding our manuscript. Your contributions and views are generally appreciated. We have incorporated the comments as you indicated within the manuscript.

**Editor’s comments**

**The concerns of the editor are outlined below:**

Please review your reference list to ensure that it is complete and correct. If you have cited papers that have been retracted, please include the rationale for doing so in the manuscript text, or remove these references and replace them with relevant current references. Any changes to the reference list should be mentioned in the rebuttal letter that accompanies your revised manuscript. If you need to cite a retracted article, indicate the article’s retracted status in the References list and also include a citation and full reference for the retraction notice.

**Author’s responses to Editor’s comments**

- We have reviewed the reference list to ensure they are complete and correct as directed by the editors.

.

**Reviewer’s comments**

Reviewer #2:

- Need to update reference. Update reference from 2019.

- Limitations of study should be add

- Rational of study need revised

**Authors’ response to reviewer 2**

-We have updated the references on pages 25 to 28 of the revised manuscript and included appropriate and current references as suggested by the reviewer.

-We have added to the limitations of the study on page 24 of the revised manuscript

-We have revised the rationale of the study on page 4 of the revised manuscript.

**Conclusions**

- We do hope that the corrections we have made in the manuscript meet the demands of the editor and reviewer.

Yours sincerely,

Kennedy Dodam Konlan
