# Supplementary material for: Burnout syndrome among healthcare workers during COVID-19 Pandemic in Accra, Ghana
Source: PLoS One. 2022 Jun 16;17(6):e0268404. doi: 10.1371/journal.pone.0268404 (PMC9202923; doi:10.1371/journal.pone.0268404)
Supplement: Attachment — Submitted filename: Response to Reviewers.docx [file pone.0268404.s008.docx]

University of Ghana

College of Health Sciences

School of Public Health

Department of Social & Behavioural

Sciences

19^th^ April, 2022

The Editor

PLos One

Dear Sir/Madam,

**RESPONSE TO MANUSCRIPT COMMENTS**

We are grateful that you considered our manuscript for review. We have provided a point by point response to each observation made by the reviewer.

**Authors’ general comments**

We are most grateful that you spent time within your important schedule to review and make insightful remarks regarding our manuscript. Your contributions and views are generally appreciated. We have incorporated the comments as you indicated within the manuscript.

**Reviewer’s comments**

Reviewer #2: Its need major revision

Tittle of study should be revised, should mentioned the city and country

Results in the abstract should be concise, only important result mentioned in abstract Rationale of study still not good, explain what benefit to society, health care workers and non clinicians, policy makers

in study setting, number of patients admitted in hospitals and OPD should explain

sample size should be calculated through EPI info software

sampling technique should be revised

Please send the questionnaire for this study for evaluation of results

**Authors’ response to reviewer 2**

-We have revised the title of the study to include the city and country as suggested by the reviewer (page 1 of the revised manuscript).

-The results in the abstract has been revised to include only relevant results (page 2 of revised manuscript).

- The rationale for the study has been revised as suggested by the reviewer (page 4 of revised manuscript).

-In the study setting, we have included the number of patients admitted in each hospital and at the OPD (pages 5 and 6 of the revised manuscript).

- We have stated the rationale for our choice of the sample size determination method we used on page 6 of the revised manuscript. Our explanation give credence to why EPI info software was not used.

- We have revised the sampling technique on page 7 of the revised manuscript.

- We have included the questionnaire that guided the study as a supporting documents. As stated in the Ethical consideration of the revised manuscript, the study was part of a bigger study titled

“Burnout, metabolic syndrome and predisposition to non-communicable diseases: A study among health workers in the Greater Accra Region of Ghana”. Part of the data in this study is what this paper is communicating.

**Conclusions**

- We do hope that the corrections we have made in the manuscript meet the demands of the editor and reviewer.

Yours sincerely,

Kennedy Dodam Konlan
